# Preventing Effect of L-Type Calcium Channel Blockade on Electrophysiological Alterations in Dentate Gyrus Granule Cells Induced by Entorhinal Amyloid Pathology

**DOI:** 10.1371/journal.pone.0117555

**Published:** 2015-02-17

**Authors:** Hamid Gholami Pourbadie, Nima Naderi, Nasrin Mehranfard, Mahyar Janahmadi, Fariba Khodagholi, Fereshteh Motamedi

**Affiliations:** 1 Department of Physiology and Pharmacology, Pasteur Institute of Iran, Tehran, Iran; 2 Neuroscience Research Center, Shahid Beheshti University of Medical Sciences, Tehran, Iran; 3 Department of Physiology, Faculty of Medicine, Shahid Beheshti University of Medical Sciences, Tehran, Iran; 4 Department of Pharmacology and Toxicology, School of Pharmacy, Shahid Beheshti University of Medical Sciences, Tehran, Iran; 5 Neurophysiology Research Center, Faculty of Medicine, Shahid Beheshti University of Medical Sciences, Tehran, Iran; 6 Neurobiology Research Center, Shahid Beheshti University of Medical Sciences, Tehran, Iran; Nathan Kline Institute and New York University Langone Medical Center, UNITED STATES

## Abstract

The entorhinal cortex (EC) is one of the earliest affected brain regions in Alzheimer’s disease (AD). EC-amyloid pathology induces synaptic failure in the dentate gyrus (DG) with resultant behavioral impairment, but there is little known about its impact on neuronal properties in the DG. It is believed that calcium dyshomeostasis plays a pivotal role in the etiology of AD. Here, the effect of the EC amyloid pathogenesis on cellular properties of DG granule cells and also possible neuroprotective role of L-type calcium channel blockers (CCBs), nimodipine and isradipine, were investigated. The amyloid beta (Aβ) 1–42 was injected bilaterally into the EC of male rats and one week later, electrophysiological properties of DG granule cells were assessed. Voltage clamp recording revealed appearance of giant sIPSC in combination with a decrease in sEPSC frequency which was partially reversed by CCBs in granule cells from Aβ treated rats. EC amyloid pathogenesis induced a significant reduction of input resistance (R_in_) accompanied by a profound decreased excitability in the DG granule cells. However, daily administration of CCBs, isradipine or nimodipine (i.c.v. for 6 days), almost preserved the normal excitability against Aβ. In conclusion, lower tendency to fire AP along with reduced R_in_ suggest that DG granule cells might undergo an alteration in the membrane ion channel activities which finally lead to the behavioral deficits observed in animal models and patients with early-stage Alzheimer’s disease.

## Introduction

Alzheimer’s disease (AD), a neurodegenerative disorder, is characterized by progressive memory impairments [1]. In AD patients, an elevated level of β-amyloid (Aβ) protein has been shown in brain regions which are involved in learning and memory such as entorhinal cortex (EC) and hippocampal formation [1]. Aβ, a 38–43 amino-acid peptide, is generated from sequential cleavage of amyloid precursor protein (APP) by β- and γ-secretase [2]. Aβ peptides play a crucial role in AD pathogenesis and aggregate to form senile plaques, a hallmark of postmortem AD brains [3–5].

An intact entorhinal-hippocampal circuit is necessary for encoding of different forms of memory [6, 7] and in AD, this network is seriously affected. Via perforant pathway, neurons in layer II and III of the EC project to all hippocampal subregions, including the dentate gyrus (DG), CA3, CA1 and subiculum [8, 9]. In the beginning stages of AD significant loss of neurons occurs in EC layer II [10]. It has been shown that neurofibrillary tangles (NFTs), a hallmark of AD, appear primarily in the EC in mild AD and spread to the adjacent regions including hippocampus and other cortical areas as the disease progresses [11]. Nevertheless, it is not entirely known which brain areas or cell types are first affected by APP/ Aβ to elicit network dysfunction in AD. AD may propagate through anatomically and functionally interconnected brain regions [11–13]. Neuronal alterations starting in the EC could spread throughout EC-hippocampal-cortical networks [14].

Cellular dysfunction and, eventually, cell death induced by Aβ is central to AD [15]. Although the precise mechanism of its toxicity is still not entirely known, Aβ induces elevated intracellular Ca^2+^ concentrations and thereby Ca^2+^ neurotoxicity and neuronal death [16–20]. Aβ causes Ca^2+^ dyshomeostasis by different ways such as increased Ca^2+^release from the intracellular source [21, 22] and/or increased Ca^2+^ influx through the plasma membrane channels including L-type voltage-gated Ca^2+^channels (L-VGCC) and N-methyl-D-aspartic acid (NMDA) receptors [20, 23]. Aging and Aβ consistently promote Ca^2+^ influx into neurons via L-type calcium channels. Moreover, soluble intraneuronal Aβ oligomers, soluble and insoluble Aβ fibrils can increase intracellular Ca^2+^, impair neuronal function, and adversely affect synaptic functions in AD [24–26].

Although DG granule cells receive massive afferents from EC via perforant pathway, there is little known about possible alterations in physiology of DG granule cells due to amyloid pathology in the EC. Harris et al. have shown that Aβ could transynaptically transfer from EC to DG and induce LTP impairment in the DG [14]. However, this result could be, in part, due to deafferentation of the DG granule cells caused by amyloid cytotoxic cell death in the EC. In this context, one of the most exciting questions is how DG granule cells, as a part of a network, respond to EC amyloid pathology? Wykes et al. have reported that APP mice show changes in intrinsic properties prior to any synaptic transmission alteration in the CA1 pyramidal neurons [27]. On the other hand, Palop et al. found that Aβ elicits aberrant excitatory activity in cortical-hippocampal networks and compensatory responses that are particularly evident in the DG [28]. In the previous work, we found that isradipine and nimodipine could improve spatial learning and memory deficit induced by microinjection of Aβ into the EC [29]. It is believed that dysfunction of the perforant pathway projection from the EC to the DG is an important contributor to the onset and progression of cognitive impairment in AD [14]. However, EC-DG interaction has not been fully assessed in experimental models of AD, and a therapeutic agent that protects the structure and function of this network has not yet been identified. Consequently, studying the underlying mechanisms leading to progressive DG amyloidopathy, dysfunction and degeneration should provide insights into AD pathogenesis. Therefore, the aim of present study was first to investigate how EC amyloid pathogenesis affects the synaptic and intrinsic electrophysiological properties in the DG granule cells. Then, the possible neuroprotective effect of L-type calcium channel blockers, isradipine and nimodipine, on intrinsic properties of DG granule cells due to EC amyloid pathogenesis was assessed by whole-cell patch clamp.

## Materials and Methods

### Ethics Statement

All experimental procedures were executed according to guidelines of the National Institutes of Health and the Iranian Society for Physiology. The study protocol was approved by the ethics committee of Neuroscience Research Center, Shahid Beheshti University of Medical Sciences under permit number 25–12–88358.

### Animals

In this study, adult male Wistar rats (180–230 g) were used. They were caged in groups of four with free access to food and water and were housed on a 12 h-light/dark cycle (light on at 07:00 h), at a temperature of 23±1°C. All efforts were made to minimize the number of animals and their suffering during the experiments.

### Drug administration

Stock solutions of human β-Amyloid 1–42 (Tocris, UK) in 0.1 M phosphate-buffered saline (PBS; pH 7.4) was prepared and aliquoted (10 μl per vial), and then stored at -70C until use. 2 μl of freshly prepared β-Amyloid (0.5 μg/μl) was used for each injection. The animals were anesthetized with intraperitoneal ketamine (100mg/kg) and xylazine (10 mg/kg). Aβ or vehicle were injected bilaterally under the stereotaxic surgery into the entorhinal cortex (AP: -5.05, L: ± 6.6 and DV: -8.2) according to the Atlas of Paxinos and Watson [30]. Using a 5-μl Hamilton syringe fitted with a 30-gauge blunt-tipped needle, injections were made over 2 min, and the needle remained in place for an additional 1 min before it was slowly retracted. After entorhinal injection, animals were implanted with cannula (8mm, 23 gauge) located 1mm above the right ventricle (AP: -0.96, L: 1.8, DV: -3.4). The cannula was fixed to the skull with a screw and dental cement. Every day, starting from the first day of the operation until the 6th day rats were given i.c.v. nimodipine, isradipine (both 30 μg/2 μl, Tocris, UK) or vehicle (DMSO, Sigma-Aldrich, USA) using a 30 gauge needle attached to a 5-μl Hamilton syringe by a polyethylene tube. The doses of nimodipine and isradipine were determined from our previous study in which both nimodipine and isradipine at 30 μg/animal have shown the most potent effects on behavioral tasks [29].

### Whole-cell patch clamp recording in slice preparation

**Acute slice preparation**. Rats were deeply anesthetized with ketamine/xylazine (intraperitoneally) and transcardially perfused with cold (4°C) modified artificial cerebrospinal fluid (ACSF) containing high sucrose and low sodium (in mM): 206 Sucrose, 2.8 KCl, 1.25 NaH2PO4, 26 NaHCO3, 10 glucose, 1 CaCl2, 2 MgSO4, pH 7.4, oxygenated with 95% O2 and 5% CO2. The animal was then decapitated and the brain rapidly removed and placed in the cold sucrose-ACSF. Transverse hippocampal slices 300 μm thick were prepared using a vibrating slicer (752 HA, Campden Instruments Ltd, UK) and transferred to a holding chamber containing modified ACSF at 32°C. After 45 minutes slices were transferred to a holding chamber and continuously superfused at room temperature with ACSF containing (in mM): 124 NaCl, 3 KCl, 1.24 NaH2PO4, 26 NaHCO3, 10 glucose, 2 CaCl2, 2 MgSO4, pH 7.4, oxygenated with 95% O2 and 5% CO2. Slices were then transferred to the stage of an upright microscope for recording (Olympus; BX 51WI).

**Whole-cell patch clamp recording**. Somatic whole-cell recordings were obtained under visual control using infrared difference interference contrast optics (IR-DIC). Dentate gyrus granule cells (DG-GCs) were visualized by infrared video imaging (Hamamatsu, ORSA, Japan) with a 40x water immersion objective. Whole cell recordings were made using Multiclamp 700B amplifier (Axon Instruments, Foster City, CA) equipped with Digidata 1320 A/D converter (Axon Instruments, Foster City, CA). For the recordings, the patch pipettes were pulled with a PC10 two-stage vertical puller (Narishige, Japan) from borosilicate glass capillary (1.2 mm O.D., 0.9 mm I.D. with inner filament). Pipettes were 3–5 MΩ when they were filled with intracellular solution. After the establishment of giga ohm, the whole-cell configuration was achieved simply by applying a brief suction. Recordings were accepted only if the series resistance was less than 25 MΩ, and if it did not vary by 20% during the experiment. In whole-cell voltage-clamp recording from acute slices, the pipettes were filled with a solution containing (in mM); 110 Cs2MeSO4, 10 HEPES, 10 EGTA, 2 MgATP, 0.3 Na2 GTP, 5 QX-314, 5 TEA-Cl, 5 4-AP and 10 Na2-Phosphocreatine at pH 7.3 adjusted with CsOH, and the osmolarity of the pipette solution was 290 mOsm. For the current clamp recording, pipettes were filled with intracellular solution (in mM) 140 K-Gluconate, 10 HEPES, 2 MgCl2, 2 Na2-ATP, 1.1 EGTA, 0.1 CaCl2 and 0.4 Na2-GTP. The pH of the internal solution was set to 7.3 by KOH, and the osmolarity was adjusted to 290 mOsm. The recordings were filtered by low-pass Bessel at 10 kHz, sampled at 20 kHz and stored on a personal computer for offline analysis. Measured electrophysiological parameters were: the frequency and amplitude of spontaneous inhibitory and excitatory post synaptic currents (sIPSC and sEPSC, respectively), resting membrane potential (RMP), action potential (AP) half width duration, AP peak amplitude, the number of APs and after hyperpolarization (AHP) amplitude.

### Histological Evaluation

Anesthetized rats were transcardially infused by PBS and then with 3% formaldehyde. Brains were rapidly removed and fixed in 30% buffered formalin, embedded with paraffin, cut into consecutive 5 μm transverse sections with a microtome and placed on poly-d-lysine-coated glass slides. TUNEL was performed by using the In Situ Cell Death Detection Kit Chemicon (Temecula, CA, USA). Tissue sections were deparaffinized in xylene, rehydrated, and in order to block the endogenous peroxidase activity immersed in 3% hydrogen peroxide. To enhance the staining, sections were treated with proteinase K solution at 37°C for 30 min after rinsing with PBS. The sections were then incubated for 60 min at 37°C with 50 μl of TUNEL reaction mixture, and then incubated for 30 min at 37°C with 50 μl of converter-POD. They were rinsed with PBS and incubated again for 10 min at 15–25°C with 50 μl of diaminobenzidine (DAB) substrate solution. Rinsing with PBS was done at the final stage. Counter staining was achieved with 0.5% methyl green. Tissue was incubated in DNase solution for 10 min at 15–25°C for positive staining. Sections were then dehydrated and cover slipped for analysis under the light microscopy. To obtain the mean percentage of the degenerated and apoptotic cells to the normal cells within the DG, the number of TUNEL positive cells was counted on three adjacent 400× microscopic images.

### Data Analysis

Data are presented as mean value ± SEM. To measure the input resistance (R_in_), 300 ms hyperpolarizing current steps (5 steps of 50 pA) were injected and corresponding membrane steady-state voltage response as a function of injected current (I-V relationship) were plotted. The statistical comparison was performed either by the unpaired two-tailed Student’s t-test, one-way or two-way analysis of variance (ANOVA), as appropriate. The rise and decay phases of the averaged sEPSCs and sIPSCs were fit with a single exponential function. Cumulative plots were analyzed by Kolmogorov-Smirnov (KS) test. Significance of correlation was determined according to the table of Pearson’s r values. Graphs were depicted using pCLAMP software and statistical tests were performed by GraphPad Prism 5.0 (GraphPad, SanDiego,CA,USA). *p* values lesser than 0.05 were accepted as significant.

## Results

### EC- amyloid pathogenesis altered excitatory and inhibitory transmission in the DG granule cells

**Fig 1:**
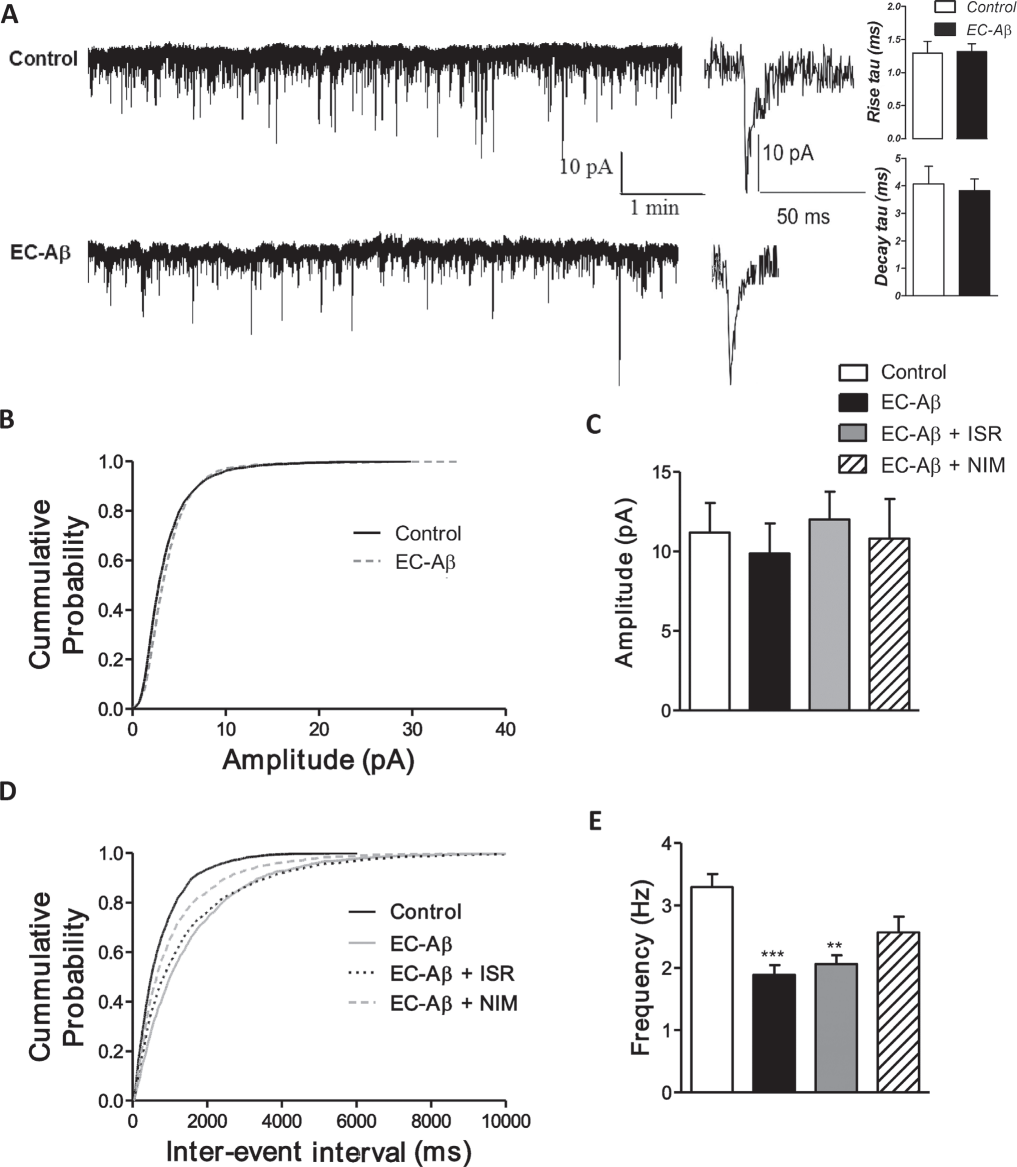
Excitatory neurotransmission onto the dentate gyrus granule cells is reduced in the granule cells from Aβ treated rats. A, Spontaneous EPSCs were less frequent in the EC-Aβ (lower trace) than control (upper trace) neurons with unaltered kinetics. Far right panel shows the analysis of the time constant of rise and decay phases and examples of sEPSCs traces of granule cells from control and Aβ treated groups which were best fit with single exponential function. Cumulative probability plot (B) and mean amplitude (C) of spontaneous EPSCs were unaltered. D, cumulative probability plots showed that spontaneous events in the EC-Aβ neurons shifted to longer inter-event intervals (IEIs) (lower frequencies). E, Daily i.c.v. treatment by nimodipine produced a significant trend (p = 0.07) towards reversing decreased sEPSCs frequency (2.569 ± 0.25 Hz, n = 6 cells/4 rats) (Fig. 1D and E). Values are mean ± SEM. **p < 0.01 and ***p < 0.001 contrasted to the control cells. Control and EC-Aβ group, n = 6 cells/ 5 rats; EC-Aβ + ISR and EC-Aβ + NIM, n = 6 cells/ 4 rats.

**Fig 2:**
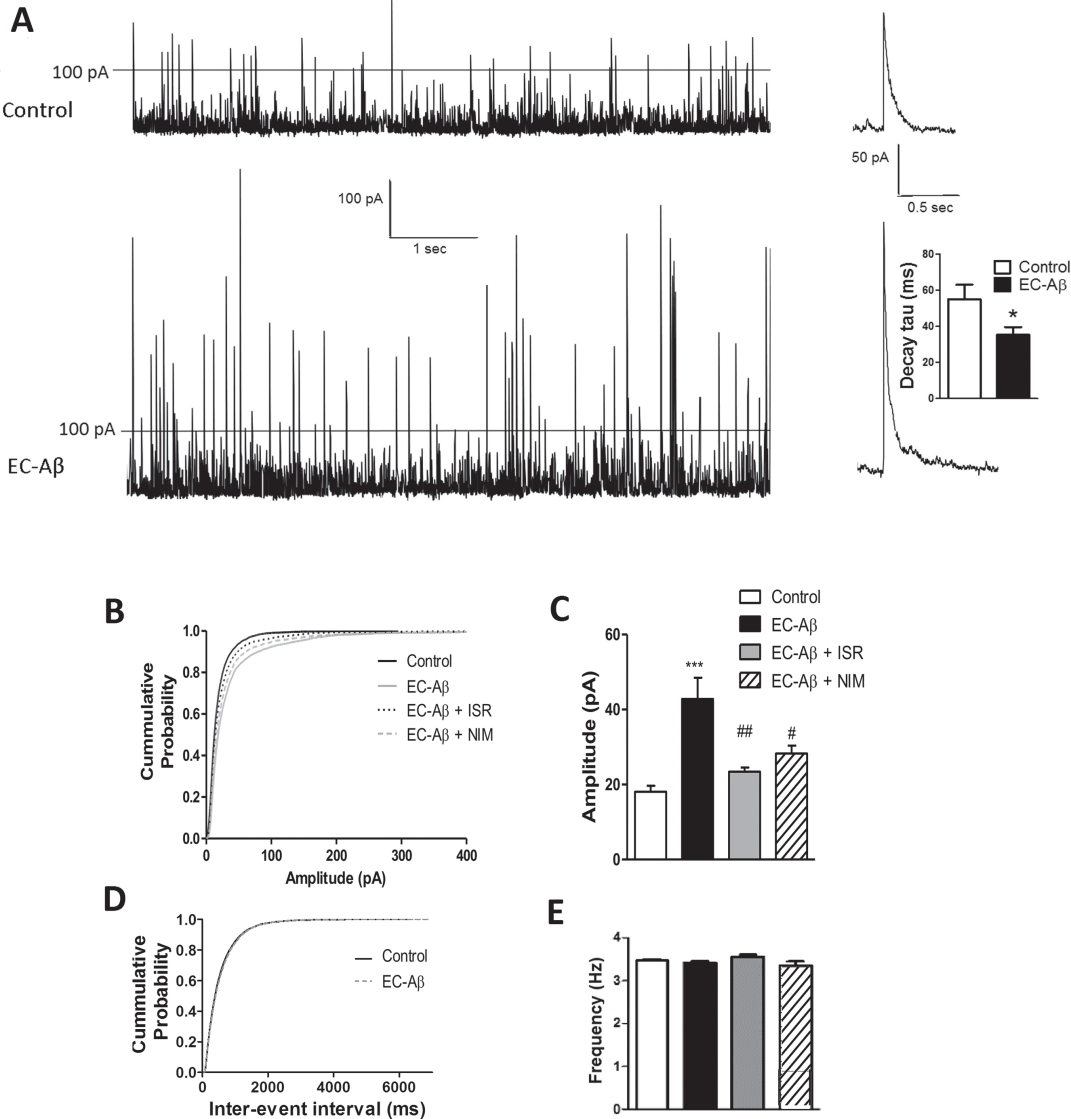
Calcium channel blockers, isradipine and nimodipine, prevent abnormalities in spontaneous IPSCs of DG granule cells caused by amyloid pathology in the EC. A, amplitude of spontaneous IPSCs increased in the EC-Aβ (lower trace) in contrast with the control (upper trace) neurons and there is a significant decrease in monoexponential decay time constant (fast inactivated) compared to the control cells (right plot, p < 0.05). B, cumulative probability plots showed that spontaneous events in the granule cells from Aβ treated rats shifted to larger amplitude (**p = 0.004 by Kolmogorov–Smirnoff test) and the change was blocked by isradipine and nimodipine. C, the amplitude of sIPSC significantly increased in granule cells from Aβ treated rats compared to the control, and isradinpine and nimodipine blocked the change. Cumulative probability plot (D) and mean frequency of spontaneous IPSCs (E) were unaltered between groups. Values are mean ± SEM. ***p < 0.001 contrasted to the control cells, ^##^p < 0.01 and ^#^p < 0.05 compared to EC-Aβ group. Control group, n = 6 cells/ 4 rats; EC-Aβ group, n = 8 cells/ 6 rats; EC-Aβ + ISR and EC-Aβ + NIM, n = 6 cells/ 4 rats.

As shown in Fig. 1, the kinetics (Fig. 1A right panel) and amplitude (Fig. 1B and C) of spontaneous EPSCs were unaffected in the EC-Aβ granule neurons. However, one way ANOVA with post-hoc Tukey test revealed that the frequency of spontaneous EPSCs in the EC-Aβ granule cells was approximately half of that in the control neurons (1.888 ± 0.15 Hz and 3.297 ± 0.20 Hz, respectively, p < 0.001). Daily i.c.v. treatment by nimodipine produced a significant near-trend (p = 0.07) towards reversing decreased sEPSCs frequency (2.569 ± 0.25 Hz, n = 6 cells) induced by Aβ (Fig. 1D and E). Aβ microinjection into the EC produced an obvious alteration in the sIPSCs amplitude (Fig. 2A). The kinetics of sIPSCs were also altered due to Aβ, so that sIPSC currents decayed more quickly than the control cells (control, 54.99 ms ± 7.97, n = 10; EC-Aβ, 35.28 ms ± 4.292, n = 12, p < 0.05) (Fig. 2A right panel). Further analysis by cumulative probability showed that amyloid pathogenesis in the EC shifted the related curve to the right, i.e. higher amplitude (p < 0.001 vs. control by Kolmogorov–Smirnoff test). Isradipine and nimodipine partially preserved sIPSCs against Aβ (Fig. 2B). The amplitude of sIPSCs were increased more than 2 folds in the granule cells in response to EC amyloid pathogenesis compared to control group (control, 18.51 ± 0.24 pA n = 6; EC-Aβ, 38.83 ± 0.83 pA, n = 8, p < 0.0001, Fig. 2C). However, in vivo treatment by isradipine and nimodipine could preserve normal amplitude of sIPSCs (EC-Aβ + ISR, 19.97 ± 0.59 pA and EC-Aβ + NIM, 20.94 ± 0.62 pA) (Fig. 2C). The cumulative analysis of sIPSC frequency showed no difference between control and Aβ treated rats (Fig. 2D and E).

### Hypoexcitability of DG granule cells in response to entorhinal cortex Aβ microinjection, and preventing role of CCBs

**Fig 3:**
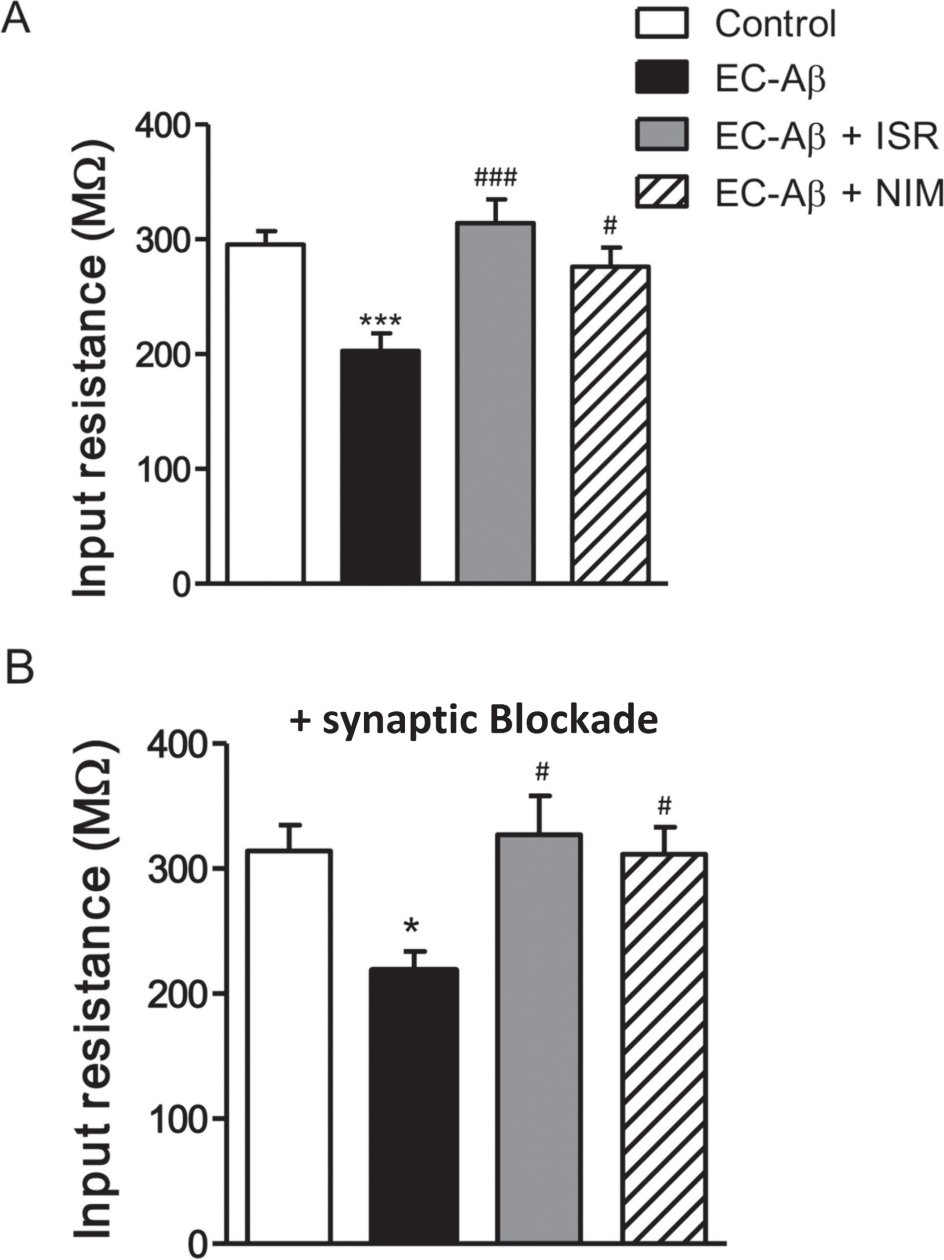
CCBs preserve normal R_in_ against EC-Aβ in the granule cells. A, R_in_ was significantly decreased in the granule cells from Aβ treated rats compared to the control group. Treatment by istradipine and nimodipine preserved R_in_ against Aβ. B, R_in_ of granule cells in presence of synaptic blockers (CNQX; 10 μM, APV; 50 μM and bicuculline; 20 μM) in different groups. Values are mean ± SEM. *p < 0.05 and ***p < 0.001 compared to the control group, ^#^p < 0.05, ^###^p < 0.001 contrasted to the EC-Aβ group. Control group, n = 15 cells/ 6 rats; EC-Aβ group, n = 15 cells/ 8 rats; EC-Aβ + ISR, n = 10 cells/ 6 rats; EC-Aβ + NIM, n = 7 cells/ 5 rats.

**Fig 4:**
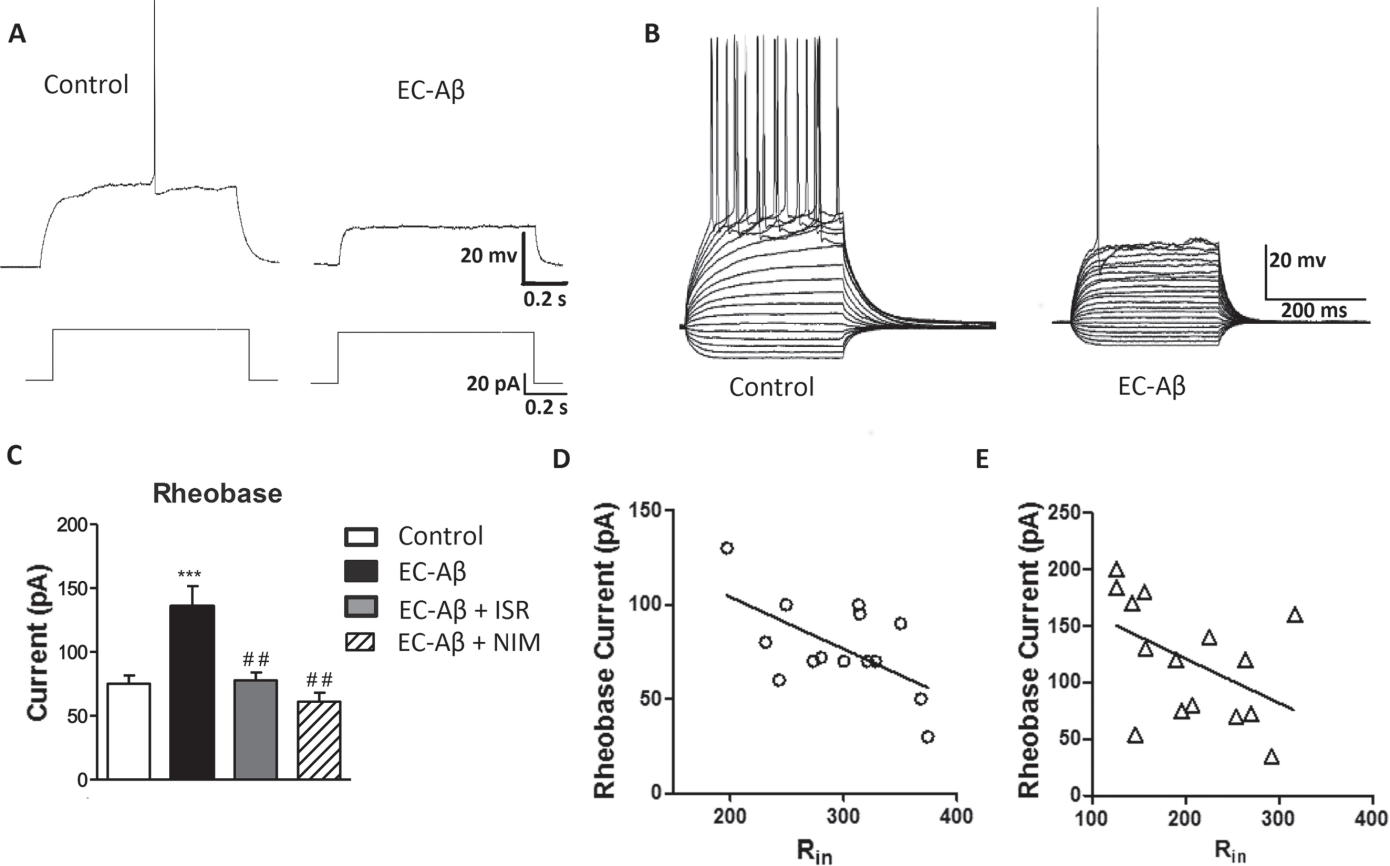
Changes of excitability of granule cells following treatment with Aβ and the protective effect of CCBs. A, a small current injection was sufficient to evoke an AP in the control granule cells, but not in the EC-Aβ cells. B, the current required to evoke minimal AP firing in EC-Aβ granule cells (right panel) evoked strong AP firing in the control cells (left panel). C, the minimal current to evoke one AP during one second (rheobase current) is increased in the EC-Aβ granule cells compared to the control group. Treatment with calcium channel blockers, isradipine and nimodipine, preserved rheobase current in the EC-Aβ granule cells. D and E, the rheobase related to the R_in_ in the control and granule cells from Aβ treated rats, respectively. Note different y and x axis scales in these panels. Values are mean ± SEM. ***p < 0.001 compared to the control group and ^##^p < 0.01 compared to the EC-Aβ group. Control group, n = 15 cells/ 6 rats; EC-Aβ group, n = 15 cells/ 8 rats; EC-Aβ + ISR, n = 10 cells/ 6 rats; EC-Aβ + NIM, n = 7 cells/ 5 rats.

Following 7 days of Aβ treatment, DG granule cells exhibited significant differences in their electrophysiological properties when compared to the control neurons. A number of electrophysiological parameters were measured by a minimal current injection to evoked one AP during 200 ms (Table 1). Aβ treatment had no significant effect on resting membrane potential, threshold, AP peak amplitude, rise slope and half width (Table 1). However, one-way ANOVA followed with Tukey’s post test revealed significantly decreased input resistance (R_in_) of the DG granule cells from Aβ treated rats (212.1 ± 17.09 MΩ) compared with the controls (295.6 ± 11.50 MΩ, p < 0.001), and treatment by israndipine (314.0 ± 20.87 MΩ) and nimodipine (276.1 ± 16.87 MΩ) could restore R_in_ to the control level (Fig. 3). On the other hand, in the presence of synaptic blockers (CNQX; 10 μM, APV; 50 μM for blocking glutamate and bicuculline; 20 μM for blocking GABA_A_ receptors), the difference between granule cells from EC-Aβ treated and control rats still remained (Fig. 3B). Ohm’s law (R = V/I) predicts that the reduced R_in_ in granule cells from Aβ treated rats should lead to a decreased excitability. Therefore, by measuring the minimal current injection required to trigger at least one action potential (rheobase) during one second (Fig. 4A), we tested this hypothesis. The rheobase current was approximately 2-fold increase in the granule cells from Aβ treated compared to the control animals, confirming the predicted decrease in excitability. In fact, the current required to evoke minimal AP firing in EC-Aβ granule cells (Fig. 4B right panel) evoked strong AP firing in control cells (left panel) (Fig. 4B and C) (control, 75.13 ± 6.3 pA, n = 15; Aβ treated, 135.7 ± 15.9 pA, n = 15, P <0.001). However, in vivo treatment with isradipine and nimodipine could preserve normal rheobase current against Aβ (isradipine-Aβ treated, 77.8 ± 6 pA, n = 10; nimodipine-Aβ treated, 61.0 ± 7 pA, n = 6) (Fig. 4C). It seems the low R_in_ caused diminished tendency to generate action potentials in the granule cells of Aβ treated rats. Moreover, there was a clear relation between the R_in_ and the rheobase (control, r = 0.59, n = 14, P <0.05; Aβ treated, r = 0.5474, n = 15, P <0.05) (Fig. 4D and E).

**Table 1:** Summary of membrane properties of DG granule cells in different groups.

Parameters	Control (n = 16 of 7 rats)	EC-Aβ (n = 19 of 8 rats)	EC-Aβ+ ISR (n = 15 of 5 rats)	EC-Aβ+ NIM (n = 14 of 4 rats)
RMP (mV)	-73.38 ± 1.653	-74.43 ± 1.135	-78.31 ± 2.580	-73.25 ± 1.887
Threshold (mV)	-39.38 ± 1.139	-39.33 ± 1.162	-40.01 ± 1.397	-39.09± 0.81
Peak Amplitude (mV)	69.72 ± 1.816	72.87 ± 2.174	68.20 ± 2.564	66.74 ± 3.13
Rise slope 10–90% (mV/ms)	73.56 ± 6.306	76.65 ± 6.002	76.15 ± 8.053	72.70 ± 6.895
Decay slope 10–90% (mV/ms)	-26.00 ± 2.828	-30.40 ± 2.851	-29.60 ± 2.941	-28.91 ± 1.865
Half width (ms)	1.713 ± 0.081	1.547 ± 0.07	1.716 ± 0.043	1.788 ± 0.1

Values are mean ± SEM. One way ANOVA showed no significant difference between different groups.

### Aβ Microinjection into the EC decreases intrinsic spiking frequency in the DG granule cells, and CCBs preserve normal firing rate

**Fig 5:**
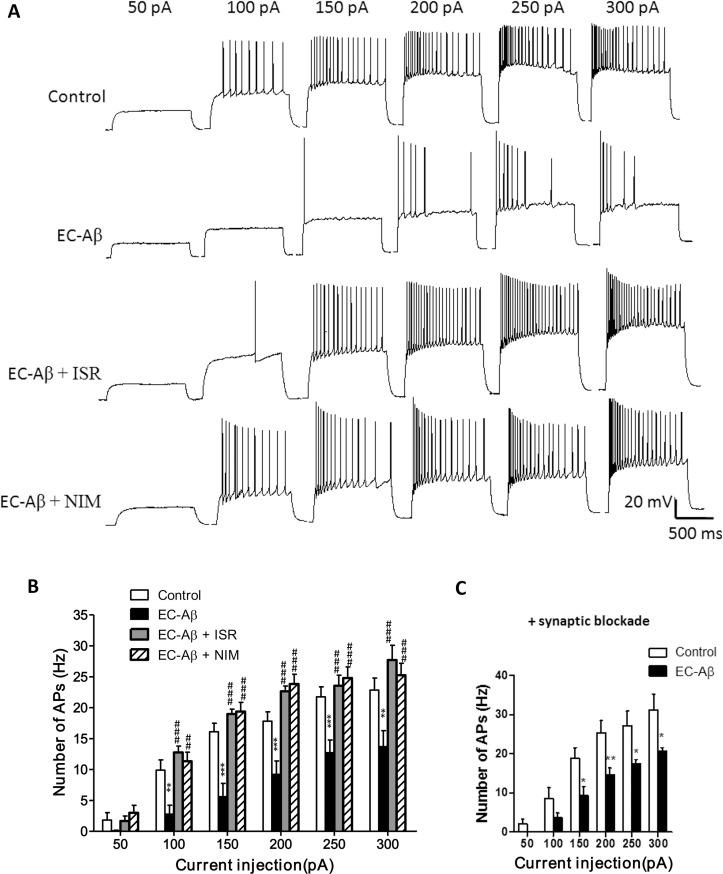
Firing properties of DG granule cells evoked by current injections. A, Aβ pathogenesis in the EC produced changes in firing pattern and decreased firing rate of DG granule cells at different current injections. Co-treatment with calcium channel blockers, isradipine or nimodipine, almost restored the normal firing pattern. B, Number of action potentials evoked at different current steps from 50 to 300 pA with intact synaptic transmission. C, Number of action potentials evoked at different current injections after synaptic transmission blockade in the presence of CNQX; 10 μM, APV; 50 μM and bicuculline; 20 μM, the difference of AP numbers still remained between the control and EC-Aβ cells. Values are mean ± SEM. *p < 0.05, **p < 0.01 and ***p < 0.001 contrasted to control cells, ^##^p < 0.01 and ^###^p < 0.001 compared to EC-Aβ group. Control group, n = 12 cells/ 6 rats; EC-Aβ group, n = 12 cells/ 8 rats; EC-Aβ + ISR, n = 10 cells/ 6 rats; EC-Aβ + NIM, n = 7 cells/ 5 rats.

We used current-clamp recordings in the granule cells to illustrate the consequence of Aβ microinjecion into the EC on their firing properties. Examples of action potential traces from the control, Aβ treated and Aβ treated along with CCBs treatment are shown in Fig. 5A. Current injections into granule cells from Aβ treated rats elicited action potentials with low frequency with an irregular firing. In fact, for the granule cells from Aβ treated rats, a larger current injection is required to produce a train of spikes than the control cells and only a few spikes were obtained. Therefore, it is not that the neuron could no longer fire repetitively but that its responsiveness has decreased. On the other hand, treatment by isradipine and nimodipine preserved almost normal firing pattern and frequency (Fig. 5A). As shown in Fig. 5B, current injection higher than 100 pA, elicited lower AP numbers in the granule cells from Aβ treated rats. However, daily microinjection of isradipine and nimodipine almost completely prevented the diminished firing rate induced by Aβ in the DG granule cells. Since Aβ induces apoptosis in the EC [29], one can expect that the DG granule cell deafferetation and aberrant synaptic input could be mirrored by abnormal firing property. This hypothesis led us to examine intrinsic properties of granule cells by excluding the impact of synaptic input using a synaptic blockers cocktail containing CNQX; 10 μM, APV; 50 μM and bicuculline; 20 μM. As shown in Fig. 5C, synaptic blockade failed to restore decreased firing rate of granule cells to the normal rate in the Aβ treated rats.

**Fig 6:**
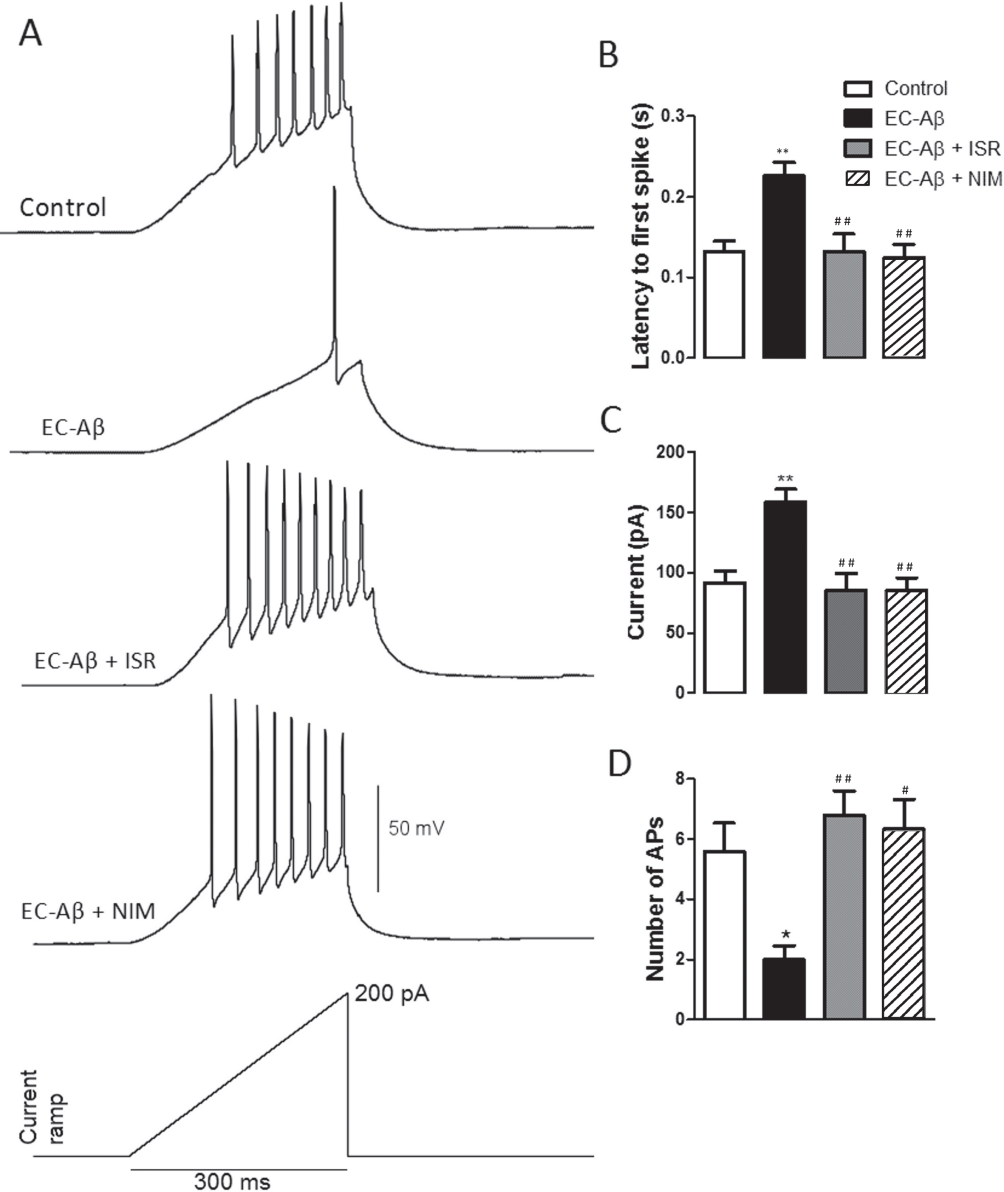
Firing properties of dentate gyrus granule cells in response to ramp currents. A, representative spike firings of DG granule cells in response to ramp current clamp. Aβ microinjection into the EC induced: a significant increase in latency of the first AP (B), a significant increase of current required to evoke the first AP (C), and a significant decrease in AP numbers (D). Treatment by isradipine and nimodipine preserved granule cells from these changes. *p < 0.05 and **p < 0.01 compared with the control cells. ^#^p < 0.05 and ^##^p < 0.01 compared to the EC-Aβ group. Control and EC-Aβ groups, n = 6 cells/ 4 rats; EC-Aβ + ISR, n = 5 cells/ 4 rats; EC-Aβ + NIM, n = 6 cells/ 4 rats.

In addition, by ramp injections of depolarizing current (200 pA, 300 ms) we also found that DG granule cells from Aβ treated rats had a lower tendency to fire APs compared with the control cells. However, isradipine and nimodipine could preserve the normal response of these cells against the Aβ (Fig. 6A). In addition, granule cells from Aβ treated rats showed a greater latency to fire the first spike compared with the control cells (control, 0.13 s ± 0.013, n = 6; EC-Aβ, 0.227 s ± 0.017, n = 6; p < 0.01). However, daily i.c.v. microinjection of isradipine (0.13 s ± 0.02, n = 5) and nimodipine (0.124s ± 0.016, n = 6) could reverse the effect of Aβ (Fig. 6B). The increased latency to the first spike was accompanied by a higher current threshold to elicit the first spike and lower firing rate in the granule cells from Aβ treated rats compared to the control cells (control, 91.80 ± 9.43 pA; EC-Aβ, 158.7 ± 10.53 pA, and control, 5.6 ± 0.92, EC-Aβ, 2.0 ± 0.44, respectively). Co-treatment Aβ with isradipine or nimodipine restored the current threshold and firing rate to the normal levels (Fig. 6C and D). Together, these data confirm that Aβ microinjection into the EC leads to hypoexcitability in the DG granule cells and CCBs could restore the normal excitability.

### Cells located in hilus are more resistant to Aβ than cells in granule layer; CCBs reduced Aβ induced TUNEL positive cells in the granule layer

**Fig 7:**
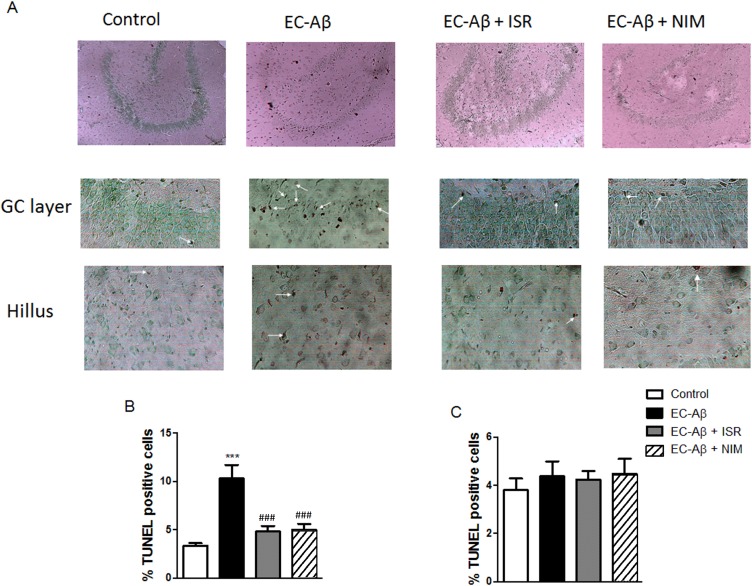
Dentate gyrus TUNEL positive cells one week after inducing amyloid pathogenesis in the EC. A, top panel indicates which region has been quantified and middle and bottom panels indicate several TUNEL positive neurons (arrows) in granule cell layer and hilus of the dentate gyrus, respectively, after Aβ microinjection into the EC. Isradipine and nimodipine could decrease TUNEL positive cells in the granule layer of Aβ treated rats. Light microscope (400x) was utilized for taking picture. B, The quantitative analysis of the data. C, no significant difference was observed between groups regarding to TUNEL-positive hilar neurons. Values are mean ± SEM. ***p < 0.001 compared to the control and ^###^p < 0.001 compared to the EC-Aβ group, n = 6.

Aβ microinjection into the EC induced significant increase of TUNEL positive cells in the granule layer of DG (control, 3.32% ± 0.29; EC-Aβ, 10.31% ± 1.38, both n = 6). However, both co-treatment with isradipine and nimodipine protected the cells in this layer against Aβ (Fig. 7B). Interestingly, cells located in hilus did not show distinctive TUNEL labeling in response to Aβ pathogenesis in the EC (Fig. 7C).

## Discussion

The main purpose of this study was to examine the effect of amyloid pathogenesis in the EC on electrophysiological properties of DG granule cells and possible neuroprotective role of L-type CCBs, nimodipine and isradipine, against Aβ. Our results showed EC amyloid pathogenesis induced hypoexcitability of DG granule cells accompanied by a reduction of R_in_, which was preserved by CCBs, isranipine and nimodipine. We also found an aberrant synaptic transmission with increased inhibition and decreased excitation input on DG granule cells which partially dampened by CCBs. An interesting finding in this study was appearance of giant sIPSC. A compensatory response might contribute to appearance of giant sIPSCs in the DG granule cells. In this line, Palop et al. demonstrate that the frequency and amplitude of miniature IPSC were increased as a compensatory remodeling of inhibitory hippocampal circuits in the hAPP mice [28]. The increased sIPSCs amplitude could underlie the lower R_in_ in the granule cells from Aβ treated rats. However, further study on intrinsic property in the presence of synaptic blockers including bicuculline as a GABA_A_ antagonist revealed that the decreased R_in_ and resultant hypoexcitability is not due to abnormal synaptic inputs on granule cells. On the other hand, the reduction of spontaneous EPSC frequency could arise from DG deafferentation and thereafter loss of synapse on the granule cells.

Indeed, the EC and its intact perforant path connections to the hippocampus are critical for learning, retrieval, and/or consolidation of spatial memory [31–34]. The EC and the hippocampus are strongly involved in spatial navigation, a function already affected in the early phases of AD. Experimental EC lesions in mice also induce synaptic loss in the DG [35–37]. EC lesions induce remodeling in the dentate gyrus granule cells [28, 37] which may change the molecular profile [38] and electrophysiological properties of these neurons. In the previous study, we showed that microinjection of Aβ into the EC decelerated reference spatial learning and impaired both learning and memory of reversal spatial task [29]. Consistently, Harris et al. reported that APP/Aβ expression in the EC caused cognitive and behavioral abnormalities such as spatial learning and memory deficits associated to abnormalities in synaptic functions and activity-related molecules in the dentate gyrus [14]. This is the first, to our knowledge, to investigate electrophysiological properties of granule cells in response to the amyloid pathogenesis in the EC. Since there are some pathological and cognitive similarities between rats with bilateral excitotoxic lesion of the EC and the early stages of AD, lesions to EC are a suitable model to simulate early phase of AD [39–41]. However, neuropathological profile due to injection of excitotoxic molecules is different from the lesions observed in AD. Therefore, injection of Aβ in EC seems to be more suitable for understanding the etiology of AD or examining neuroprotective effect of candidate molecules against Aβ pathogenesis in the early stage of AD [41]. Most of the residential neurons in hilus are inhibitory interneurons which contain GABA. In the histology experiment, we found that neurons located in hilus did not show TUNEL positive staining in either control or Aβ treated animals indicating their resistance in face of EC amyloid pathology. However, because interneurons located in this region do not have massive direct connections with perforant path, it could be suggested that these cells may be far enough from being influenced, functionally or directly, by Aβ in EC. Consistently, it has been reported that unlike molecular layer of DG, Aβ deposition is minimal or absent in the hilus and mossy fiber pathway in the EC-APP transgenic mice. On the other hand, it has been reported that interneurons of DG because of their high content of calcium-binding proteins such as parvalbumin or calretinin are resistant in different neurodegenerative conditions [42, 43].

Synaptic inputs and intrinsic membrane characteristics are two key factors determining neural excitability [44, 45]. Changes in either synaptic activities or membrane intrinsic properties affect the output of the neuron [46]. Our data showed that granule cells from Aβ treated rats possessed a strongly reduced R_in_, which is an important factor in postsynaptic signal integration. No such decrease in R_in_ has been reported in any of the many earlier patch-clamp studies of granule cells using other AD animal or cell models [27, 47–49]. However, Haghani et al reported a decrease of R_in_ in the CA1 pyramidal cells due to Aβ microinjection into the deep frontal cortex [50]. There are many reports indicating that Aβ induces hypoexcitability. In the culture, granule cells showed decreased excitability along with increased AHP amplitude in response to incubated Aβ [48]. In parallel, there are also some reports indicating decreased excitability of CA1 pyramidal neurons in response to Aβ [50, 51]. In contrast, some studies have reported little or no change in the intrinsic excitability of hippocampal or cortical neurons in the face of a significant amyloid load [52, 53].

There are two possible mechanisms to discuss how EC amyloid exposure may lead to alterations in DG granule cells excitability. First, as a compensatory response to EC amyloidopathy, an alteration of molecular profile in the DG may occur which could finally lead to functional changes in DG cells. Consistent with this suggestion, Harris et al. demonstrated that overexpression of APP in EC elicited alterations in calbindin and synaptic activity-related (Fos and NPY) proteins in the DG [14]. Such a molecular alterations may lead to hypoexcitability of DG granule cells in response to microinjection of Aβ into EC in our work. Second, alteration in excitability could also result from direct actions of presynaptically released Aβ on post-synaptic membranes. In this regard, it has been shown that APP/Aβ could transaxonally transported from EC to DG [14, 54]. If so, DG granule cells could be exposed to Aβ which may directly induce synaptic and intrinsic alterations in these neurons. In this line, Yun et al. found that direct application of oligomeric A𝛃 (1–42) to DG granule cells could decrease their excitability [48].

In the voltage clamp experiment, we observed significant increase of sIPSC amplitude along with decreased frequency of sEPSC in the granule cells of Aβ treated rats. Since one determinant for R_in_ is synaptic input [55], we examined R_in_ and firing rate of granule cells in presence of a cocktail of postsynaptic receptor blockers. However, this cocktail failed to increase R_in_ (Fig. 3B) and firing rate (Fig. 5C) to the normal level in the granule cells from Aβ treated rats. Regarding negative correlation between R_in_ and membrane conductance, over-expression of membrane ion channels such as potassium channel could underlie low R_in_ in the DG granule cells in response to Aβ pathology in the EC, which remains to be investigated. There are many reports indicating Aβ causes increased current and upregulation of different subtypes of potassium channels such as A-type K channels [56, 57]. Since we found a decrease in R_in_ and hypoexcitability, our results is in line with an increase of potassium current. In this line, Cameron et al. reported that potassium conductance has a major role in determination of R_in_ [58]. Young et al. found decreased R_in_ and excitability, associated with upregulation of inward rectifier K^+^ (K_ir_) channels conductance in the DG granule cells in a temporal lobe epilepsy kainate model [59].

We found L-type calcium channel blockers, isradipine and nimodipine, could almost prevent aberrant passive and active electrophysiological properties and to a less extent imbalanced synaptic transmission in the DG granule cells induced by amyloid pathogenesis in the EC. In this study, CCBs were microinjected into the lateral ventricle. Their improving effect could arise from protecting EC neurons against direct exposure to Aβ, then preventing subsequent functional alterations in DG cells. In the previous work, we found that CCBs efficiently preserved EC neurons against Aβ [29]. In parallel, Anekonda et al. showed that CCBs protect mice MC65 neuroblastoma cells against Aβ neurotoxicity [20]. However, part of their protective effect could be mediated directly by preserving normal homeostasis of DG cells. It has been shown that nimodipine could reduce the post-burst AHP of CA1 pyramidal neurons in vitro [60], enhance the firing rate of hippocampal pyramidal neurons in vivo [61] and attenuate the learning deficit in aged animals [62]. Many reports have provided direct evidence for the involvement of L-type Ca^2+^ channels in Aβ-induced neuronal toxicity in neurons or cell lines [16, 63, 64]. In fact, it is widely proposed that neuronal Ca^2+^ homeostasis is compromised in AD [18, 24, 65]. Many studies have focused either on channels that can directly allow Ca^2+^ influx into the cells including L-type Ca^2+^ channel, or channels that control neuronal excitability, which are closely coupled to Ca^2+^ signaling, largely through control of voltage dependent Ca^2+^ channel gating [66]. A number of studies have shown that Aβ can disrupt neuronal Ca^2+^ homeostasis by inducing influx of extracellular Ca^2+^ into the neuronal cytoplasm [20, 67, 68]. Furthermore, Aβ induces specific increases in the quantity and activity of L-type Ca^2+^ channels [69]. Neuronal L-type Ca^2+^ channels are known to be involved in translating synaptic activity, into alterations in gene expression and in neuronal cell death [70–72]. The mechanism underlying reduced R_in_ and hypoexcitability in the granule cells following EC amyloid pathogenesis and protecting effect of CCBs in face of Aβ is not clear. One possible mechanism is that calcium dyshomeostasis due to Aβ may lead to increase activation and/or expression of potassium channels and resultant diminished R_in_ and hypoexcitability. On other hand, in vivo treatment by CCBs may prevent Ca^2+^ dyshomeostasis and therefore preserves normal potassium currents and cell firing against Aβ.

In summary, amyloid pathogenesis in the EC results in altered intrinsic cellular properties of DG granule cells which persist even after synaptic blockade. Increased latency to fire the first AP and low tendency to produce a train of APs are in favor of A-type potassium current in the granule cells from Aβ treated rats. CCBs almost completely restore normal cellular properties in granule cells which are accompanied by protection of granule cells from apoptosis. Further studies are needed to illustrate the precise mechanism in which whether CCBs rescue DG granule cells from EC amyloidopathy by protecting EC neurons from apoptosis, or acting on different subtypes of membrane ion channels in the DG.
